# Improving and sustaining delivery of CPT for PTSD in mental health systems: a cluster randomized trial

**DOI:** 10.1186/s13012-017-0544-5

**Published:** 2017-03-06

**Authors:** Shannon Wiltsey Stirman, Erin P. Finley, Norman Shields, Joan Cook, Rachel Haine-Schlagel, James F. Burgess, Linda Dimeff, Kelly Koerner, Michael Suvak, Cassidy A. Gutner, David Gagnon, Tasoula Masina, Matthew Beristianos, Kera Mallard, Vanessa Ramirez, Candice Monson

**Affiliations:** 1 National Center for PTSD and Stanford University Department of Psychiatry and Behavioral Sciences, 795 Willow Road, Menlo Park, CA 94025 USA; 2 The University of Texas Health Science Center at San Antonio, Department of Psychiatry and Medicine, 7703 Floyd Curl Dr, San Antonio, TX 78229 USA; 3 Divisional Psychologist Occupational Health and Safety, Royal Canadian Mounted Police, 4225 Dorchester, Westmount, QC Canada; 4 Department of Psychiatry, Yale University, 950 Campbell Avenue, West Haven, CT 06516 USA; 5 San Diego State University, 5500 Campanile Dr, San Diego, CA 92182 USA; 6 Boston University School of Public Health, Department of Health Law, Policy and Management, Boston, MA 02215 USA; 7 Evidence-Based Practice Institute, 3303 S Irving Street Seattle, Seattle, WA 91844 USA; 8 Suffolk University, 73 Tremont Street, Boston, MA 01331 USA; 9 National Center for PTSD, VA Boston Healthcare System, 150 S. Huntington Ave, Boston, MA 02130 USA; 10 Ryerson University, 350 Victoria Street, Toronto, ON M5B 2K3 Canada; 11 National Center for PTSD and Palo Alto Veterans Institute of Research, 795 Willow Road, Menlo Park, CA 94025 USA; 12 Center for Healthcare Organization and Implementation Research (CHOIR), Department of Veterans Affairs Boston Healthcare System, Boston, MA USA; 13 South Texas Veterans Health Care System, 7400 Merton Minter St, San Antonio, TX 78229 USA; 14 Boston University School of Medicine, 72 E Concord St, Boston, MA 02118 USA

**Keywords:** Implementation, Sustainability, Delivery, CPT, Fidelity, Learning collaborative, EBPs

## Abstract

**Background:**

Large-scale implementation of evidence-based psychotherapies (EBPs) such as cognitive processing therapy (CPT) for posttraumatic stress disorder can have a tremendous impact on mental and physical health, healthcare utilization, and quality of life. While many mental health systems (MHS) have invested heavily in programs to implement EBPs, few eligible patients receive EBPs in routine care settings, and clinicians do not appear to deliver the full treatment protocol to many of their patients. Emerging evidence suggests that when CPT and other EBPs are delivered at low levels of fidelity, clinical outcomes are negatively impacted. Thus, identifying strategies to improve and sustain the delivery of CPT and other EBPs is critical. Existing literature has suggested two competing strategies to promote sustainability. One emphasizes fidelity to the treatment protocol through ongoing consultation and fidelity monitoring. The other focuses on improving the fit and effectiveness of these treatments through appropriate adaptations to the treatment or the clinical setting through a process of data-driven, continuous quality improvement. Neither has been evaluated in terms of impact on sustained implementation.

**Methods:**

To compare these approaches on the key sustainability outcomes and provide initial guidance on sustainability strategies, we propose a cluster randomized trial with mental health clinics (*n* = 32) in three diverse MHSs that have implemented CPT. Cohorts of clinicians and clinical managers will participate in 1 year of a fidelity oriented learning collaborative or 1 year of a continuous quality improvement-oriented learning collaborative. Patient-level PTSD symptom change, CPT fidelity and adaptation, penetration, and clinics’ capacity to deliver EBP will be examined. Survey and interview data will also be collected to investigate multilevel influences on the success of the two learning collaborative strategies. This research will be conducted by a team of investigators with expertise in CPT implementation, mixed method research strategies, quality improvement, and implementation science, with input from stakeholders in each participating MHS.

**Discussion:**

It will have broad implications for supporting ongoing delivery of EBPs in mental health and healthcare systems and settings. The resulting products have the potential to significantly improve efforts to ensure ongoing high quality implementation and consumer access to EBPs.

**Trial registration:**

NCT02449421. Registered 02/09/2015

## Background

Thousands of public sector clinicians have been trained to deliver evidence-based psychotherapies (EBPs) due to recent mandates and investments in implementation [1, 2]. Patients treated during EBP training programs experience substantial symptom improvement [3–5]. Despite this progress, efforts to implement and sustain EBPs in these systems face significant challenges. One finding is that penetration (integration of a practice within a service setting and its subsystems) is low [6, 7]. Veteran Affairs clinicians rarely cite established contraindications as reasons not to offer EBPs; [8, 9] instead, they cite challenging symptom profiles, a need for more consultation, and clinic-level barriers [10, 11]. A second challenge is that research suggests that fidelity (adherence to prescribed elements of treatment and competence/skill of delivery) [12] may be low when these interventions are implemented [13, 14]. Challenges in implementing EBPs lead to adaptation without systematic efforts to understand the impact on symptoms and other outcomes [15, 16]. While some adaptation and latitude in EBP delivery may be appropriate [17], effective [18–21], or promote implementation [22], other adaptations result in discontinuation of core elements [15], integration of non-evidence-based strategies [13, 23], and worse outcomes [24, 25]. In addition to these challenges, local capacity can impact sustainability and quality of delivery. A significant minority of clinician trainees do not meet established EBP competency criteria but deliver EBP protocols or EBP elements in their practice [11, 13, 15]. Turnover and organizational context can also impact sustained EBP delivery [26, 27]. Patient access and clinical outcomes may suffer as a result [23, 24, 28–30]. Thus, in the context of routine care, improvement, rather than maintenance, [31] of clinical outcomes may be an appropriate goal. Finally, while EBPs are cost-effective [32], the budget impact of supporting implementation can be a barrier [33–35].

Failure to provide EBPs to those with PTSD can have a significant public health impact. Consequences of inadequate treatment include risk of suicide, overuse of healthcare, work absenteeism, reduced productivity, unemployment, and family disruption [36, 37]. Cognitive processing therapy (CPT), which has been implemented in at least eight countries and in mental health systems throughout the world, has a strong evidence base and is effective with a variety of patient populations [19, 20, 38–42]. The current study will aid in identifying strategies to promote sustainability of CPT and improve patient-level outcomes, thereby informing efforts to implement EBPs for psychiatric disorders more broadly.

Researchers have identified serious gaps in knowledge regarding best practices for improving and sustaining EBPs in routine care [43–45]. There have been few, if any, experimental investigations of strategies to sustain EBPs. Few studies on sustainability have assessed a full range of the key outcomes, such as patient-level mental health outcomes [46]. To address these gaps and identify effective strategies for sustaining EBPs in routine care, we seek to compare two promising yet different implementation strategies (ISs), fidelity-oriented consultation, and continuous quality improvement.

### Proposed interventions

Existing research and theory suggest two contrasting ISs, one oriented to fidelity and the other to mutual adaptation and quality improvement. These strategies have potentially different mechanisms, costs, and relative benefits that could impact their fit in differing contexts.

### Fidelity-oriented learning collaborative (FID)

Studies show that without consistent follow-up or ongoing support of clinicians to promote fidelity after initial training, the training effects quickly dissipate [47, 48]. Although a recent meta-analysis demonstrated no overall link between observer-rated fidelity and symptom change across a range of treatments and disorders, when analyses were conducted to avoid the potential temporal confound between fidelity and symptom change and establish the temporal precedence of fidelity, two aspects of fidelity, adherence to the protocol and competence (skill of delivery) in early CBT sessions were associated with subsequent decreases in depression [49]. Later research suggested that fidelity to the key aspects of CPT, as opposed to prescribed session elements, was associated with symptom change [50]. Other studies demonstrated patient-level benefits of fidelity-oriented support for EBPs as compared to general professional development-oriented support [51–53]. A FID strategy has been shown to lead to clinician achievement of benchmark levels of fidelity [54]. We would thus expect FID to impact clinical outcomes through CPT fidelity as a mechanism of change. Fidelity support also appears to impact other implementation outcomes, most notably sustained EBP capacity, delivery [55], and support activities [56]. Fidelity support has also been associated with lower staff turnover, which improves workforce capacity [53].

### Continuous quality improvement-oriented learning collaboratives (CQI)

Organization-level barriers to implementation are commonly found in routine care settings and may impact sustainability [4, 26, 57, 58]. In our study on CPT training consultation in Canada, [59] in Texas, and in the US VA [10, 11], clinicians cited organization-level challenges to delivering EBPs for PTSD and hesitance about offering EBPs, due in part to a perceived lack of fit between the treatment and the patients [9]. These problems may contribute to the low rates of penetration [6, 7]. The Dynamic Sustainability Framework (DSF) suggests that the dynamic “fit” between an intervention and its delivery context is critical to sustainability [60]. It rejects the assumption that deviation from the protocol leads to decreased benefit and advocates for mutual adaptation and continuous refinement of EBPs in real world contexts [60]. CQI, identified in the DSF as a facilitator of sustainability, has been used successfully in healthcare and mental health settings [61, 62] and has guided EBP adaptation [63]. CQI also fosters learning organizations, which are more likely to improve care and innovate [64]. We therefore expect that the mechanisms by which CQI impacts clinical outcomes are EBP adaptation and functioning as a learning organization.

While high-level leadership support is strong, and structural and policy level changes and investments have been made to support implementation within many systems, [2] the individual clinicians who must ultimately deliver treatments have identified idiosyncratic challenges at local levels. The locally oriented, clinician-led approach of CQI-oriented learning collaboratives [61] (LCs) may facilitate sustainability, but this possibility remains unexamined [65]. On the other hand, greater time, cost, and personnel burden [66, 67] may be a barrier to CQI, and whether the CQI process and resulting adaptations lead to more effective treatment and implementation is unknown. To answer these questions, we will conduct a mixed method, type-III hybrid design, which allows simultaneous study of ISs, their mechanisms, and clinical outcomes [68].

### Summary of the proposed project

Clinics in three mental health systems that have implemented CPT for PTSD will submit baseline session recordings and patient data for at least two patients (*n* = 192) before random assignment to either 12 months of FID (*n* = 16 clinics, 48 clinicians, 192 patients), or CQI (*n* = 16 clinics, 48 clinicians, 192 patients). Outcomes will include patient self-reported PTSD symptom outcomes (primary); independent fidelity ratings; and penetration, adaptation, and capacity to deliver CPT [46, 69]. We will also investigate engagement in, credibility and costs of, and satisfaction with each IS [mechanisms by which the interventions impact patient outcomes] as well as contextual factors that may impact sustainability. The mixed method design will include qualitative methods for a richer understanding of the process and outcomes for each of the ISs [70]. This study will capitalize on infrastructure created for a previous trial comparing EBP consultation strategies [59] as well as existing infrastructure in the participating mental health systems and strong collaborative relationships and experience with each participating mental health system. The study has the potential to advance implementation science beyond observational studies of EBP sustainability by providing much-needed information on the effectiveness of interventions to promote long-term EBP implementation.

## Methods

### Summary

We plan to randomize clinics and associated clinicians in three mental health systems that have implemented CPT for at least 2 years into one of two conditions: CQI-LC or FID. Implementation outcomes will be assessed. ISs will be compared over the course of the 1-year intervention phase and a 1-year follow-up. Mechanisms of action and budget impact will also be explored. A phased rollout of the ISs will allow baseline data to serve as within and across subject comparison data. Potential individual and contextual influences on sustainability will also be assessed through surveys and interviews to elucidate how these factors may impact CPT delivery and IS engagement and outcomes.

### Recruitment

Participating clinics and clinicians will be drawn from the three mental health systems: the VA Canada Operational Stress Injury (OSI) National Network and affiliated clinics (VAC), the United States VA system, and the State of Texas (TX). Each of these systems has implemented CPT in the past several years, with standardized training across the networks which includes an initial workshop followed by 6 months of consultation. As Table 1 indicates, the systems are diverse in terms of clinician backgrounds, patient populations, and financing.

**Table 1:** Characteristics of participating mental health systems

Source	No. of clinicians available for recruitment	No. of clinics (planned/ available)	Clinic type/population	Clinician degrees	Payment/reimbursement	Penetration
VA Canada OSI Network (VAC)	134 OSI clinicians and contracted community clinicians, 65% received CPT provider status	11/38	VAC clinics and community-based/veterans and civilians	M.A., PhD/PsyD	Government veteran benefits, private insurance, self-pay for civilians	~60% of eligible patients (by self-report)
United States VA system (VA)	2996 trained, 73% reached provider status	11/138	VA hospitals and clinics/veterans	PhD/PsyD, MSW, MD	Typically VA benefits, TriCare	112–20% eligible patients [6]
Texas (TX) Community MH Clinics	253 trained/53% reached CPT provider status	10/115	Civilians and rural veterans	MSW, LICSW, LPC, CART, MA, PhD/PsyD	Medicaid, VA Tricare, self-pay, private insurance	To be assessed

We plan to recruit and enroll an average of 3–4 clinicians per clinic across 32 clinics over the first 2 years of the study.

### Inclusion/exclusion criteria

We will recruit VAC and VA clinics, TX, and Canadian community clinics that participated in CPT training and have at least 4 clinicians eligible to participate. Clinics are defined as work units under the same supervisor or service line within an organization. Clinicians and supervisors who maintain a caseload will be eligible to participate in the study if they (1) are CPT-trained and provide psychotherapy to individuals with PTSD, (2) are willing to record therapy sessions, provide work samples for baseline assessment, and complete baseline surveys, and (3) have internet access. We considered only enrolling clinicians who had achieved a certain level of CPT adherence (e.g., “provider status”) but recognized that stakeholders need to understand how to improve EBP delivery among *all* clinicians who deliver EBPs within the system, including the 23–47% of clinicians (Table 1) who receive training but do not achieve provider status. Their inclusion allows assessment of ISs’ ability to improve capacity to deliver high quality CPT.

Eligible patient participants are Veterans or non-Veterans (1) diagnosed by their clinicians to have current PTSD, with a minimum PTSD checklist (PCL) score of 33 (based on the most current validation and cutoff scores that indicate a likely PTSD diagnosis for the PCL-5) [71] 2) who have not previously received CPT, and (3) are willing to consent to completing symptom measures and have their sessions audio recorded and reviewed. Patients are permitted to continue other psychotherapies if the treatment is not specifically focused on PTSD. Ineligible patients are those ineligible for CPT based on the state of research evidence as follows: (1) current uncontrolled psychotic or bipolar disorder, (2) [past month] substance dependence requiring daily use or detoxification (eligibility can be revisited if individuals enter recovery), (3) imminent suicide/homicide risk requiring immediate intervention, and (4) cognitive impairment precluding therapy engagement (e.g., significantly impaired memory or attention).

### Randomization and timeline

Randomization will occur at the clinic level. Given the relatively small number of clinics, a stratified randomization procedure will be used to match the ISs (after baseline) on size (# of clinicians in each clinic) and system. The statistician will create a block for each combination of covariates and assign each clinic to the appropriate block. Simple randomization will occur for clinics within each block. As illustrated in the timeline, ISs will occur in three waves, and within each wave, clinics will be randomized further into immediate or delayed (6 months) start. This will facilitate a comparison with no IS [72] using symptom and session data collected from the baseline phase of the delayed start group. Administrative data from before and during the IS phase will facilitate further comparison of symptom change for patients seen before vs. during ISs. During baseline and to keep the delayed start group engaged, we will offer webinars to introduce study technology and provide general updates on CPT research findings (with no guidance for actual practice). Incentives for engagement in baseline include written fidelity feedback from one baseline session after the IS phase starts, clinician and clinic-level gifts for providing baseline data, and CE credits for webinars.

### ISs

*PracticeGround*, an internet-based EBP training and learning community platform, will be used as the platform for IS meetings in this study. It has capacity for usage metrics and provision of CE credits. Previous research demonstrated its effectiveness as a platform for EBP training [73, 74]. A full range of materials for CPT online support (e.g., webinars, video clips, presentations, and handouts on CPT-related topics) have been integrated into PracticeGround.

Each IS cohort will include a maximum of eight clinicians across up to two clinics within the same system. After a 2-hour web-based learning (for CQI) or booster (for FID) sessions, they will meet biweekly for the first 3 months, then monthly over the last 9 months of the [12-months] active intervention phase, with between-meeting message board correspondence and communication within individual clinics. We will review recordings to assess adherence to the FID or CQI manual and differentiation of ISs with a checklist based on a review of CQI elements and the key IS elements identified in the manuals [75] and will track correspondence within each condition to compare communication.

### Fidelity-oriented consultation (FID)

FID will be led by a CPT expert using structured format based on the effective standard CPT consultation model [4, 5, 76]. One hour meetings will consist of fidelity (adherence and competence) feedback based on discussion of cases and session audio review during the meeting, with guidance on challenges to CPT fidelity, e.g., how to use CPT without deviation from the manual. Participants will also review CPT training modules to improve fidelity. Facilitators will be certified CPT consultants. PDSA cycles will not be used.

### CQI learning collaborative (CQI)

CQI facilitators have been trained in CQI and LC strategies by study team members who have experience in facilitating CQI-oriented LCs. The framework for the CQIs is based on the Institute for Healthcare Improvement’s Breakthrough Series collaborative model for quality improvement [77], which has been used successfully in mental health [61]. Prior research indicated that in-person learning sessions do not add a significant value relative to associated costs [78], so initial learning sessions will be trainings in CQI principles that will occur on PracticeGround. Action periods take place between subsequent hour-long Web-based meetings, allowing time for each team to implement change ideas identified during meetings and study the effectiveness of those change ideas using the Plan-Do-Study-Act (PDSA) cycle of learning. Examples of goals might include increasing CPT engagement, improving effectiveness for individuals with particular symptom profiles, or advocating for more frequent sessions within the clinic. Consistent with the CQI model, non-clinicians who are the key to producing change can participate in the CQI meetings to facilitate change.

### Hypotheses and assessment strategy

We propose to assess the impact of CQI and FID on a variety of the key implementation outcomes with the following hypotheses:

1. Patient symptom improvement
  - Our primary outcome, change in PCL-5 scores, non-inferior for CQI as compared to FID
  - Outcomes for ISs will be superior to outcomes for patients seen during the no intervention phase
  - The ISs will impact change in symptoms through different mechanisms (increased fidelity for FID [30, 79], CPT adaptation, and development of learning organizations for CQI) [64]
2. Cost, as measured by budget impact, will be greater for CQI, which requires increased personnel burden (management involvement and activities between meetings) [61], than FID.
3. Fidelity (adherence and competence) will be greater for FID, which targets fidelity [54], than CQI [60].
4. Adaptation (# of changes to CPT) will be greater for CQI than FID due to planned adaptations in CQI [60].
5. Penetration (% of eligible patients who receive CPT) will be greater for CQI than FID, as CQI targets barriers to patient enrollment and retention and allows adaptations to improve fit [60].
6. Workforce capacity (% of clinicians who receive CPT provider status) will be greater for FID, which is intended to improve skills and promote adherence [54], the benchmarks for quality-rated provider status.
7. Organizational capacity (learning organization status and implementation climate) will be greater after CQI, which facilitates development of learning organizations [64] and reduces barriers to implementation [60, 80].

### Patient measures

To examine hypotheses 1A and B, we will use the posttraumatic stress disorder checklist (PCL-5), a validated 20-item self-report measure that assesses the 20 DSM-5 symptoms of PTSD [71] that patients routinely complete in CPT. Each item is measured on a 5-point Likert scale. These are recorded in VA clinical records and can be extracted at the clinic and patient level. VAC collects the PCL-5 in their secure, Web-based Client-Reported Outcome Monitoring Information System (CROMIS), which is based on the OQ outcomes monitoring system [81]. For CPT patients, PCLs are done at pre-treatment, at every CPT session, at post-treatment and 3-months post-treatment, for a total of 12–15 administrations.

### Additional patient measures

Patient demographics and diagnoses will be collected from clinicians and used as covariates in analyses. Patients will also complete The Outcome Questionnaire-45 (OQ-45), a validated, 15 min measure of functioning and other key outcomes of interest (symptoms, interpersonal problems, social role functioning, and quality of life) that are of interest in mental health [82, 83] (baseline, mid- and post-treatment) and the Patient Health Questionnaire (PHQ-9) [84] to assess symptoms of depression (at every session). They will complete a post-treatment Client Satisfaction Questionnaire (CSQ-8) [85] to assess satisfaction with treatment at the end of treatment.

### Cost (budget impact)

To test hypothesis 2 and lay the groundwork for future, more comprehensive economic evaluations, we will use a framework for best practices for budget impact analysis [86].

### Clinician CPT fidelity assessment

#### Observer-rated fidelity measures

We will assess fidelity for hypothesis 3 and as a potential mechanism of change (hypothesis 1C) via observer ratings of a randomly selected subset of session recordings. The CPT fidelity measure (Nishith and Resick Cognitive Processing Therapy Fidelity Scale. Unpublished Manuscript) examines clinicians’ adherence and competence to specific CPT interventions prescribed in each session. Clinicians are rated on their adherence to the protocol (i.e., the degree to which they performed a particular task, on a 0–2 Likert-type scale) and competence in delivery of these elements (rated on a 7-point, Likert-type scale). Sessions will be continuously uploaded by clinicians to a secure server (using procedures that were successfully used with geographically dispersed clinicians in our consultation study) [62, 87] and randomly selected for rating to minimize bias that may occur if clinicians were instructed to only provide sessions at designated time points. Rater agreement will be maintained through ongoing training, and raters will rate at least 15% of the sessions to assess reliability.

#### Self-reported adherence measure (secondary measure of fidelity)

As a routine aspect of documentation, at every session, clinicians will complete a 5–8-item adherence checklist for “unique and essential” CPT elements that are embedded in required VA template clinical notes and which will also be embedded in the CROMIS/OQ platforms.

### Adaptation

To assess adaptation (hypothesis 4), we will use a framework and coding system of modifications and adaptations made to EBPs [88] that has previously been applied to interview and observation data on CPT adaptation [26]. Session recordings will be independently rated to identify modifications.

### Penetration, clinician satisfaction, LC engagement, and CPT activity reporting (P&S)

As in our consultation study, at baseline, 4, 8, and 12 months, and at the follow-up, clinicians will complete a survey on CPT activity. Questions will include number/proportion of PTSD cases who received CPT since the last assessment, number of CPT sessions delivered, satisfaction with the IS over the past month; frequency of non-study consultation or training; contextual adaptations; and confidence in CPT delivery. To assess the validity of self-reported penetration, detailed interviews with a subset of clinicians will assess eligibility of each patient with PTSD on the caseload and identify CPT elements utilized with each.

### Capacity

To measure workforce capacity to deliver CPT (hypothesis 6), we will examine the proportion of clinicians in each clinic who qualify for a more advanced form of provider status, quality-rated CPT provider status (QRCPS) at each assessment. QRCPS is awarded to clinicians who have conducted CPT with at least three patients in the past year, and who achieve an average of two (out of three points) or higher adherence score and an average of four (out of six, indicating good) competence scores on a randomly selected session.

To assess organizational capacity and contextual factors (hypothesis 7), we will use two measures.

The Learning Organization Inventory (LOI) [80] is a psychometrically valid assessment of learning environment, concrete learning processes, and whether leaders reinforce learning. Given the potential for CQI to foster development of learning organizations, LOI will be assessed at two additional times for our analysis of potential mechanisms. We will also assess organization- and clinician-level constructs that have been associated with implementation success in prior research [27, 89]. The Implementation Climate Assessment (ICA) [89] will be used as an additional measure of capacity.

### Potential moderators (exploratory)

Additional measures will be used to explore organization- and clinician-level moderating effects on the impact of ISs and/or use of EBPs, they will be administered at the baseline, 4-, 6-,12-, and 24-months timepoints (also to examine change over time) of the study [90].

The Organizational Social Context (OSC) measures climate, culture, and work attitudes. It is psychometrically sound [91] and predicted sustainability in prior research [27].

The Implementation Leadership Scale (ILS) [92] is a brief measure of (1) proactive, (2) knowledgeable, (3) supportive, (4) perseverant leadership with excellent internal consistency, convergent, and discriminant validity.

The Clinician Demographic Characteristics and Experience Questionnaire (CDCEQ) will be administered at baseline to assess relevant clinician demographic information and experience.

The Perceived Characteristics of Intervention Scale (PCIS) [93] is a brief survey that assesses innovation characteristics first identified by Rogers that are hypothesized to influence adoption and sustainability [93].

### Interviews and qualitative strategy

We will also interview a subset of clinicians and administrators to contextualize and extend findings from the quantitative data collection. We will sample from each clinic at baseline. At post-LC and follow-up, a purposive sampling strategy will be utilized to ensure representation across systems and to capture perspectives of clinicians and administrators who experience different outcomes within each IS condition (e.g., low vs. high penetration, fidelity, and CPT utilization). The interviews will assess multilevel influences on engagement in ISs and on CPT delivery. The interview guide will be based on the tenants of the DSF [60] and the Consolidated Framework for Implementation Research [94].

#### Analytic strategy

We will examine descriptive statistics and distribution of all variables. Outcome variables will be transformed as needed if data violate assumptions of normality. We will evaluate effectiveness of randomization by comparing baseline measures of the key variables and demographics using χ^2^ and *t* tests.

##### Aim 1

The first aim is to examine the impact of two LC interventions for sustained EBP delivery on the use and effectiveness of CPT. We will test hypothesis 1A (non-inferiority of FID relative to CQI) by examining [95] whether the difference in change between the groups after the ISs is complete (12 months) and at follow-up is smaller than a predetermined clinically reliable difference (i.e., the non-inferiority margin “delta”).

To estimate change, we will use multilevel regression (i.e., growth curve analyses, mixed effect regression, hierarchical linear modeling), which offers numerous strengths for analyzing change over time for nested data, including efficiency in handling missing data, powerful and accurate estimation procedures adjusting for clustering, and modeling flexibility (e.g., allows for the inclusion of continuous or categorical, time invariant or time varying, covariates and predictors). This approach is extremely robust to missing data and considered “state of the art” for analyzing unbalanced data sets due to dropouts and loss to follow-up [96].

A three-level model will be evaluated to test the impact of IS on change in PTSD. Time (since pre-treatment) will be entered as a level-1 predictor to estimate change in PCL-5 scores. Level 2 will be included to account for the individual assessments nested within patients. Since randomization occurred at the clinic level, IS (dummy coded variable) will be entered as a level-3 predictor to examine the impact of IS on change over time in PTSD symptoms. Given the small number of systems, system will be a level-3 (clinic level) variable to assess and adjust for its influence. We will examine the influence of potentially significant covariates (e.g., baseline PCL-5, VA vs. community; veteran status, other clinician, or patient characteristics/diagnoses) and include in analyses if necessary.

To examine hypothesis 1B (that both ISs will be superior to no intervention), we will conduct piecewise multilevel growth modeling, which will allow us to estimate separate trajectories for each phase of the study by condition. This approach is being increasingly used in randomized controlled trials to more clearly elucidate change across multiple phases [97].

To further compare the impact of the ISs to no intervention (hypothesis 1B), we will use PCL and diagnostic data extracted from VAC’s CROMIS and the VA records. We will separately examine administrative PCL data for all patients with PTSD seen by VAC and VA clinicians through CROMIS and VA’s clinical CPT templates and clinical reminder data, both of which have capacity for clinic and clinician-level linkage and data extraction.

Hypothesis 1C. Different mechanisms of change will be evident for the two ISs. We hypothesize that FID will impact outcomes through increased fidelity and that CQI will impact outcomes through adaptation and development of learning organizations. Lagged regression modeling is a useful way to examine temporal precedence between contemporaneous time-varying phenomena, where a causal effect of variable X1 on X2 is established when X1 at time 1 significantly contributes to the behavior of X2 at time 2 while controlling for the effect of X2 at time 1 [98, 99]. To evaluate temporal precedence, lagged multivariate multilevel models will be evaluated [100]. Multivariate models will allow for the inclusion of two outcomes in the same model [100]. Each of the outcomes will be predicted by the level of that outcome at the previous time point (i.e., autoregressive effect, e.g., PTSD at time 2 will be predicted by PTSD at time 1) and the other variable (observer-rated fidelity, adaptation, or LOS score) in the model at the previous assessment occasion (i.e., cross-lagged path PTSD will be predicted by the proposed mechanism and vice-versa). We will also account for overall increases and decreases in the model by including time as a predictor of each outcome.

##### Sample size justification and power calculations

We require a sufficient sample to detect non-inferiority based on a clinically significant change of 5 points on the PCL [101, 102]. Calculations of a design effect (measure of how the design effects the standard error of the parameters) [103, 104] accounted for clustering. We computed intraclass correlations (ICC) with repeated observations of CPT patients at the clinic (*ρ* = 0.12), clinician (*ρ* = 0.16), and patient (*ρ* = 0.38) level in our prior research in Canada and VA, yielding a design effect of up to 2.32 [83]. Power to detect a clinically meaningful difference on PTSD symptom scores over time, taking the design effect into account, is .80 in a two-sided test, using repeated measures, with an alpha-level of 0.05 and a standard deviation (sd) of 7 points (based on prior research), for a sample size during the IS phase of 288 patients (144 per condition) within 32 clusters. In the proposed study, we expect to randomize 32 clinics, with an average of three clinicians per clinic and at least four patients per clinician during both the IS and follow-up phase. For each patient, PTSD symptoms will be measured at nine time points, as it is typical to assess symptoms frequently in CPT (baseline, every other session, post-treatment, and follow-up). To very conservatively account for potential patient-level attrition and missing data during treatment, we estimated an average of four observations per patient. Furthermore, we considered the longer study length, rates of turnover in each system, attrition rate in previous studies, balanced with participation in an active intervention, system support, and incentives, to anticipate substantial, but not unmanageable, attrition. Full participation in the IS and follow-up would yield 384 patients and at least 1536 observations, and thus, we could see a clinician attrition rate of 33% while remaining adequately powered to detect non-inferiority of CQI, and superiority of the ISs as compared to the baseline/non-IS phase data (an additional 2 patients per clinician; up to 192 patients). This projection exceeds the rate of attrition seen in our prior studies in these systems and is sufficient to test for non-inferiority on OQ measure as a secondary analysis, as the OQ-45 has an RCI of 14 (sd = 16) [105]. Finally, the sample will allow for a stable estimate of a fidelity based on the recommended *G* = .70–.80 level of dependability) [106].

3.I.5 Testing of additional hypotheses and exploratory analyses. To compare the budgetary impact of each IS (hypothesis 2), a budget impact analysis (BIA) [107] will be conducted to estimate the cost to the system of implementing and sustaining each IS. Results will be interpreted in conjunction with the findings for hypothesis 1A. If evidence of non-inferiority is found, the less expensive IS may be warranted, but significant differences in favor of the more costly IS might justify the added expense.

Hypotheses 3–7 involve continuous outcome data and will employ a similar multilevel regression strategy as described in section 3.I.1. We will also examine interactions between these variables and ISs to assess for a moderating effect on patient outcomes and will similarly explore potential moderating effects of attitudes (EBPAS) and social context (OSC) and leadership (ILS) on the key outcomes of interest. We will explore whether the system impacts the effects of the ISs by testing interactions between system, IS, and time.

##### Aim 2

The second aim is to assess each IS’s acceptability and perceived fit in diverse mental health systems.

##### Qualitative and mixed method analytic strategy

We will use interviews, qualitative strategies, and surveys to understand barriers and facilitators of the ISs and to CPT. Qualitative and quantitative data will be integrated to facilitate a fine-grained understanding of processes and characteristics that influence clinicians’ use and perceptions of CPT as well as fit and effectiveness of ISs within each system. A priori codes will be based on the DSF and Aarons et al.’s model of EBP implementation and sustainability [34, 85]. Using NVIVO 11 software, we will code transcripts from different stakeholders and through consensus, and identify and operationalize additional emerging codes. We will identify the central themes by diversity and triangulation across data sources, the frequency with which specific influences were identified, and attention to the key decision points and interactions described in data sources. Survey data on contextual factors will be examined in conjunction with qualitative data for the purposes of contextualization, validation, and triangulation [70, 108] to understand how contextual factors influence experience with CPT and ISs.

## Discussion

Little research has been conducted to investigate different strategies to promote the sustained use of interventions. This study will contribute to a growing literature on sustainability by employing a mixed method approach to examining two different strategies across different mental health systems, offering several innovations. The application of a hybrid-type-III design [and data on the budgetary impact of each IS] [35, 47] allows the study of relative advantages of ISs and comparison of clinical outcomes in diverse care settings. Information about budget impact, time, personnel burden, perceived fit, and the key implementation outcomes will provide meaningful guidance for policy and decision-making on future implementation efforts. Penetration, fidelity, adaptation, capacity, and patient outcomes are the key implementation outcomes [69] that have not been objectively examined in prior research [46]. Patient outcomes have not been routinely assessed in mental health sustainability research [46] 3). Fidelity and adaptation have rarely been objectively assessed, and prior efforts to objectively evaluate fidelity have lacked full participation. This study is also one of the first examinations of the tenets of the Dynamic Sustainability Framework (DSF) [60], which encourages CQI and adaptation to improve sustainability and clinical outcomes. Many previous implementation studies have focused on either initial implementation, changing clinician behavior [51, 105] and skill or on promoting change at the leadership, organization, and system level [34]. Testing clinic-based CQI will allow us to examine the extent to which clinician-led, data-driven mutual adaptation can facilitate sustainability. We anticipate that this research will yield several products: a manual and toolkit for effective IS(s), papers describing implementation and patient-level outcomes, an examination of mechanisms of influence for each implementation strategy, papers reporting on multilevel predictors of sustained CPT implementation, a mixed method comparison of system, clinic, and clinician experiences of ISs in three mental health systems and comparisons of clinician vs. observer-generated data on fidelity and adaptation.

## Abbreviations

BIA: Budget impact analysis
CDCEQ: Clinician Demographic Characteristics and Experience Questionnaire
CPT: Cognitive processing therapy
CQI: Continuous quality improvement
CROMIS: Client Reported Outcome Monitoring Information System
DOD: Department of Defense
DSF: Dynamic Sustainability Framework
EBP: Evidence-based practice
EBPAS: Evidence-based practice attitudes scale
FID: Fidelity
ICA: Implementation climate assessment
ICC: Intraclass correlation
ILS: Implementation Leadership Scale
IS: Implementation strategy
LC: Learning collaborative
LOI: Learning Organization Inventory
LOS: Learning Organization Survey
MHS: Mental health system
OSC: Organizational social context
OSI: Operational stress injury clinic
PCIS: Perceived Characteristics of Intervention Scale
PCL: PTSD checklist
PDSA: Plan, Do, Study, Act
PE: Prolonged exposure
PTSD: Posttraumatic stress disorder
QRCPS: Quality-rated CPT provider status
TX: Texas
US: United States
VA: Veterans Affairs
VAC: Veterans Affairs Canada
